# Scribble and α-Catenin cooperatively regulate epithelial homeostasis and growth

**DOI:** 10.3389/fcell.2022.912001

**Published:** 2022-09-21

**Authors:** Yunxian Huang, Jinghua Gui, Satu-Marja Myllymäki, Kallol Roy, Tambet Tõnissoo, Marja L. Mikkola, Osamu Shimmi

**Affiliations:** ^1^ Institute of Biotechnology, University of Helsinki, Helsinki, Finland; ^2^ Institute of Computer Science, University of Tartu, Tartu, Estonia; ^3^ Institute of Molecular and Cell Biology, University of Tartu, Tartu, Estonia

**Keywords:** cell polarity, mammalian cells, *Drosophila melanogaster*, epithelial cells, homeostasis, adherens junction

## Abstract

Epithelial homeostasis is an emergent property of both physical and biochemical signals emanating from neighboring cells and across tissue. A recent study reveals that Scribble, an apico-basal polarity determinant, cooperates with α-Catenin, an adherens junction component, to regulate tissue homeostasis in the *Drosophila* wing imaginal disc. However, it remains to be addressed whether similar mechanisms are utilized in vertebrates. In this study, we first address how α-Catenin cooperates with Scribble to regulate epithelial homeostasis and growth in mammalian cells. Our data show that α-Catenin and Scribble interact physically in mammalian cells. We then found that both α-Catenin and Scribble are required for regulating nuclear translocation of YAP, an effector of the Hippo signaling pathway. Furthermore, ectopic Scribble suffices to suppress YAP in an α-Catenin-dependent manner. Then, to test our hypothesis that Scribble amounts impact epithelial growth, we use the *Drosophila* wing imaginal disc. We show that Scribble expression is complementary to Yorkie signal, the *Drosophila* ortholog of YAP. Ectopic expression of full-length Scribble or Scribble Leucine Rich Region (LRR):α-Catenin chimera sufficiently down-regulates Yorkie signal, leading to smaller wing size. Moreover, Scribble LRR:α-Catenin chimera rescues *scribble* mutant clones in the wing imaginal disc to maintain tissue homeostasis. Taken together, our studies suggest that the association of cell polarity component Scribble with α-Catenin plays a conserved role in epithelial homeostasis and growth.

## Introduction

Formation of correct size and shape of organs in animal development requires precise control of tissue growth (Hafen and Stocker, 2003; Hariharan, 2015). During organogenesis, epithelial cells within a multicellular tissue sense the mechanical cues both across the tissue and from abutting tissue. Mechanical cues can promptly trigger local biochemical responses such as actomyosin assembly, thus regulating cell behaviors (Rizzelli et al., 2020).

The Hippo pathway was originally identified in *Drosophila melanogaster*, where its loss results in tissue overgrowth and hippopotamus-shaped adult phenotypes (Huang et al., 2005; Hansen et al., 2015). Hippo signaling is regulated by a variety of upstream signaling pathways, including cell polarity regulation, levels of F-actin, tension within the actin cytoskeleton, and cell attachments (Dupont et al., 2011; Fernandez et al., 2011; Sansores-Garcia et al., 2011; Gaspar and Tapon, 2014; Hansen et al., 2015). In vertebrates, Yes-associated protein (YAP), an effector of the Hippo pathway, is phosphorylated by Large tumor suppressor 1/2 (Lats1/2), resulting in retention in the cytoplasm or in degradation. Otherwise, YAP moves into the nucleus in a complex with its partner Transcriptional coactivator with PDZ-binding motif (TAZ) to regulate YAP-dependent transcription and growth (Totaro et al., 2018; Zheng and Pan, 2019). In mammalian cell culture, cells that are cultured at high density have low YAP activity, whereas cells cultured at low density or are physically stretched have high YAP activity, indicating that YAP and TAZ act as mechanotransducers (Zhao et al., 2007; Dupont et al., 2011; Benham-Pyle et al., 2015). The key molecules in Hippo signaling are evolutionarily conserved: in *Drosophila*, phosphorylation of Yorkie (Yki), a homologue of YAP, by Warts (Wts, Lats1/2 homologue in *Drosophila*) promotes cytoplasmic localization of Yki, thus reducing Yki-dependent transcription and growth (Justice et al., 1995; Hansen et al., 2015).

Interactions of Hippo pathway components with cell-cell junctions and the cytoskeleton suggest potential mechanisms for the regulation of the pathway by cell-cell contact. In both mammalian and *Drosophila* epithelial cells, E-Cadherin (E-Cad)-mediated cell-cell adhesion at adherens junctions (AJs) appears to play a central role in mediating a mechanical circuit that integrates adhesion, contractile forces and biochemical signaling (Rubsam et al., 2017; Kale et al., 2018). The core components of the AJs are E-Cad and their binding partners α-, β-, and p120-Catenins. As a transmembrane protein, E-Cad forms intercellular bridges through trans homophilic interactions of the extracellular domains. The stability of such interactions depends on intercellular binding partners, of which β-Cat is the most significant. By interacting with E-Cad, β-Cat recruits α-Cat, which serves as a platform for actomyosin filaments that stabilize AJs and regulate cell-autonomous morphogenesis or affect adjacent cells through AJs (Takeichi, 2014; Sumi et al., 2018). Individual epithelial cells are constantly exposed to centripetal force generated by contraction of the circumferential actomyosin ring, which is sensed by E-Cad and α-Cat as mechano-transducers (Yonemura et al., 2010; Borghi et al., 2012; Buckley et al., 2014; Rauskolb et al., 2014; Alegot et al., 2019).

In epithelial morphogenesis, establishing cell polarity plays a key role in maintaining tissue growth and homeostasis (Hariharan and Bilder, 2006; Rodriguez-Boulan and Macara, 2014). Apico-basal cell polarity establishes distinct membrane domains along the apico-basal axis of epithelial cells to segregate specific cellular functions to the apical or basolateral regions (Achilleos et al., 2010; Bonello et al., 2019). Three key modules, including the Crumbs (Crb) and partitioning defective (PAR) complexes, as well as the Scribble (Scrib) module, have been shown to be involved in establishing apico-basal polarity (Thompson, 2013; Lang and Munro, 2017; Bonello and Peifer, 2019). In *Drosophila*, Scrib, together with discs-large (Dlg) and lethal giant larvae (Lgl), is localized at the basolateral cell membranes and is enriched at the septate junctions (SJs), the functional counterpart of mammalian tight junctions (TJs), to sustain the basolateral compartment (Bilder and Perrimon, 2000; Sharifkhodaei et al., 2019). Loss of *scrib* or its partners leads to compromised cell polarity and neoplasia (Bilder, 2004; Zeitler et al., 2004). Importantly, the deregulation of Scrib affects Hippo pathway signaling (Cordenonsi et al., 2011; Yang et al., 2015; Bonello and Peifer, 2019).

We previously showed that intercellular control of apico-basal polarity plays a unique role in regulating growth of epithelial tissues in the *Drosophila* wing imaginal disc (Gui et al., 2021). When *scrib* hypomorphic mutant (*scrib* ^*5*^) cells are surrounded by wild type cells, apico-basal polarity in mutant cells is non-autonomously restored. Therefore, tissue homeostasis is preserved with restrained Yki activity. Furthermore, conditional RNAi knockdown of *scrib* leads to loss of apico-basal polarity autonomously as well as non-autonomously, with hyperactivation of Yki signal. Importantly, these phenotypes were largely restored by the Scrib-Leucine Rich Region (LRR):α-Cat chimeric protein. Our previous data showed that Scrib and α-Cat can form a complex, protein distributions of Scrib and α-Cat are mutually regulated, and α-Cat fused with SJ component Kune, but not E-cad, can partially restore loss of *scrib* phenotypes (Gui et al., 2021). These facts suggest that α-Cat functions with Scrib to regulate epithelial homeostasis.

In this study, we first addressed how interactions between α-Cat and Scrib are conserved in vertebrates by using mammalian tissue culture cells. We further confirmed that both α-Cat and Scrib are key regulators of YAP localization. We then revealed that ectopic expression of Scrib together with α-Cat sufficiently suppresses the nuclear translocation of YAP when cells are maintained at low density in culture. By using the *Drosophila* larval wing imaginal disc, we found that spatiotemporal control of Scrib appears to be crucial for Yki activity. Our data also shows that ectopic expression of full-length Scrib or Scrib-LRR:α-Cat chimera modulates Yki activity, resulting in different tissue sizes. Finally, our data suggest that α-Cat interactions with Scrib are crucial and sufficient for epithelial homeostasis and growth *in vivo*. Thus, our findings indicate that α-Cat and Scrib cooperatively regulate epithelial homeostasis and growth in both vertebrates and invertebrates.

## Materials and methods

### Cell culture and production of recombinant proteins

A375 cells were cultured in DMEM containing 2 mM Glutamine, 10% Fetal Bovine Serum (FBS), 100 IU penicillin, and 100 μg/ml streptomycin. HEK 293T cells were maintained in DMEM medium containing 10% FBS, 100 IU penicillin, and 100 μg/ml streptomycin.

Cells were transfected with HiFugene transfection reagent (Promega) according to the manufacturer’s protocol. Three days after transfection, cells were collected and cell lysates were subjected to immunoprecipitation (IP) using the GFP-Nanotrap Kit (Chromotek) according to the manufacturer’s instructions. Cells were lysed in IP lysis buffer (25mMTris-HCl pH 7.4, 150 mM NaCl, 1% NP-40, 1 mM EDTA, 5% glycerol) on ice for 30 min. The supernatants obtained were used in Western blotting and subsequent IP. Western blotting was conducted as previously described (Gui et al., 2016). All biochemical data shown are representative of no less than three independent assays.

Cells were cultured on coverslips for 3 days, then fixed with 4% paraformaldehyde for 20 min at room temperature. Cells were permeabilized in PBT for 15 min at room temperature, then stained with antibodies or proximity ligation assay reagents. For the Duolink proximity ligation assay, the blocking buffer, PLA probe and detection reagent kit were obtained from Sigma-Aldrich. Cells were blocked in Duolink Blocking buffer for 1 h at 37 °C. Primary antibodies were diluted in Duolink Antibody Diluent buffer, and cells were incubated overnight at 4°C. Experimental samples were incubated with mouse anti-human α-Cat and rabbit anti-human Scrib, whereas negative controls were incubated with mouse anti-human α-Cat and rabbit anti-Myc as control. The following morning, cells were incubated with PLUS PLA probe anti-rabbit and MINUS PLA probe anti-mouse for 2 h at 37°C. Ligation and amplification steps of the PLA were performed using the Duolink *in situ* Detection Reagents Red kit according to the manufacturer’s instructions. Images were then obtained by confocal microscopy.

### RNA interference

The following shRNAs were synthesized by Sigma-Aldrich:

Lentivirus-mediated shRNA: Based on the protocols provided by Addgene, lentivirus constructs carrying shRNA against human Scrib or α-Cat were generated and delivered to cultured cells upon packaging into replication-deficient lentiviral particles to generate knockdown clones. The packaging and envelope plasmids, including psPAX2 and pMD2. G, were obtained from Addgene. The shRNA transfer vector plasmid against Scrib (TRCN0000004458) and the shRNA transfer vector plasmid against α-Cat (TRCN0000234533) were obtained from Sigma. Control cells were transduced with lentiviral particles carrying pLKO.1 Non-Target shRNA Control Plasmid (Sigma).

### Fly genetics

*Ex-lacZ* (#44248), *nub-GAL4* (#25754), *tubP-GAL80* ^*ts*^ (#7017) and *scrib* ^*2*^ (#41775) were obtained from the Bloomington *Drosophila* Stock Center (BDSC). Scrib:GFP (CA07683) was obtained from Fly Trap projects (Morin et al., 2001). αCat:GFP (#115921) was obtained from Kyoto Stock Center. Fly stocks were maintained at 25°C unless otherwise mentioned. To induce MARCM clones, larvae were heat-shocked for 2 h at 37°C 3 days after egg laying, and samples were collected 2 days after heat shock. Larvae and adults for conditional knockdown mediated by *nub-GAL4* were maintained at 27°C after egg laying.

### Full genotypes

Figure 3A, B: *Ex-lacZ*.

Figure 3C: *Scrib:GFP*.

Figure 3F *αCat::GFP*.

Figure 4A–C: *nub-GAL4/Ex-lacZ; tub-GAL80* ^*ts*^ */+, or nub-GAL4/Ex-lacZ, UAS-scrib. FL:Myc; tub-GAL80* ^*ts*^ */+, or nub-GAL4/Ex-lacZ, UAS-LRR:Myc; tub-GAL80* ^*ts*^ */+, or nub-GAL4/Ex-lacZ, UAS-LRR:α-Cat:Myc; tub-GAL80* ^*ts*^ */+*

Figure 5B–D: *hsFLP/+; tub-GAL4, UAS-mCD8-GFP/+; FRT82B, tub-GAL80/FRT82B scrib* ^*2*^ *, or hsFLP/+; tub-GAL4, UAS-mCD8-GFP/UAS-scribFL:Myc; FRT82B, tub-GAL80/FRT82B scrib* ^*2*^ *, or hsFLP/+; tub-GAL4, UAS-mCD8-GFP/UAS-LRR:Myc; FRT82B, tub-GAL80/FRT82B scrib* ^*2*^ *, or hsFLP/+; tub-GAL4, UAS-mCD8-GFP/+; FRT82B, tub-GAL80/FRT82B, or hsFLP/+; tub-GAL4, UAS-mCD8-GFP/UAS-dLRR:Myc; FRT82B, tub-GAL80/FRT82B scrib* ^*2*^ *, or hsFLP/+; tub-GAL4, UAS-mCD8-GFP/UAS-LRR:αCat:Myc; FRT82B, tub-GAL80/FRT82B scrib* ^*2*^ *, or hsFLP/+; tub-GAL4, UAS-mCD8-GFP/UAS-α-Cat:Myc; FRT82B, tub-GAL80/FRT82B scrib* ^*2*^

### Antibodies, chemicals and immunohistochemistry

Larvae were fixed in 3.7% formaldehyde at room temperature for 20 min, after which wing imaginal discs were dissected. The following primary antibodies were used: rat anti-DE-Cad (1:50) (Developmental Studies Hybridoma Bank (DSHB)), mouse anti-GFP (1: 5,000 for Western blotting, Millipore), rabbit anti-Myc (1:100, Santa Cruz Biotechnology), mouse anti-β-Galaxtosidase (1:500, Promega), mouse anti-human YAP (1:50), rabbit anti-cleaved-Dcp-1 (1:200, Cell Signaling Technology), mouse anti-human α-Cat (1:50, Santa Cruz Biotechnology), rabbit anti-human Scrib (1:50, Abcam), and rabbit anti-human YAP (1:50, Cell Signaling Technologies).

Secondary antibodies (1:200) were as follows: goat anti-mouse IgG Alexa 488, goat anti-mouse IgG Alexa 568, goat anti-mouse IgG Alexa 647, goat anti-rabbit IgG Alexa 488, goat anti-rabbit IgG Alexa 568, goat anti-rat IgG Alexa 488 and Alexa488-conjugated phalloidin (all from Thermo Fisher Scientific).

### Imaging and image analysis

Fluorescent images were obtained with Zeiss LSM700 and Leica SP8 upright confocal microscopes. All confocal immunofluorescent images were processed and analyzed with ImageJ/FIJI. The images were the composite of a stack with projection of max intensity unless otherwise specified. Quantification of nuclear/cytoplasmic signal (ratio N/C) was obtained from YAP intensity in the nuclei and cytoplasm segmented in ImageJ/FIJI (Schindelin et al., 2012).

### DNA constructs

To generate human *scrib* cDNA construct, we obtained full-length (FL) and truncated human *scrib* cDNA from PLK45 (#37250, Addgene). Human α-Cat cDNA was from α-Cat1-906 (#24194, Addgene). α-Cat, FL, truncated Scrib or LRR:αCat with C-terminal Myc tag were cloned into the PEF1-His-myc vector. The constructs encode the following human Scrib protein amino acids: FL, 1-1630; LRR, 1-701; PDZ1/2, 702-976; PDZ3/4, 977-1216; CT, 978-1630. All Scrib fragments were cloned into EcoRI and Xbal sites; α-Cat was cloned into EcoRI and NotI sites. The chimeric construct was generated by inserting α-Cat CDS into the C-terminus of LRR before the stop codon. α-Cat with C-terminal GFP was cloned into pEGFP-N1 at EcoRI and KpnI sites.

### Statistics

All experiments were carried out independently at least three times. Statistical analyses were performed using GraphPad Prism software (v.9.0.2, GraphPad). The number for all quantified data is indicated in the figure legends. Data are means ±95% confidence intervals (CIs). Statistical significance was calculated by the two tailed *t*-test method and Dunnett’s test after one-way ANOVA.

## Results

### Scrib interacts with α-cat in mammalian cells

**FIGURE 1:**
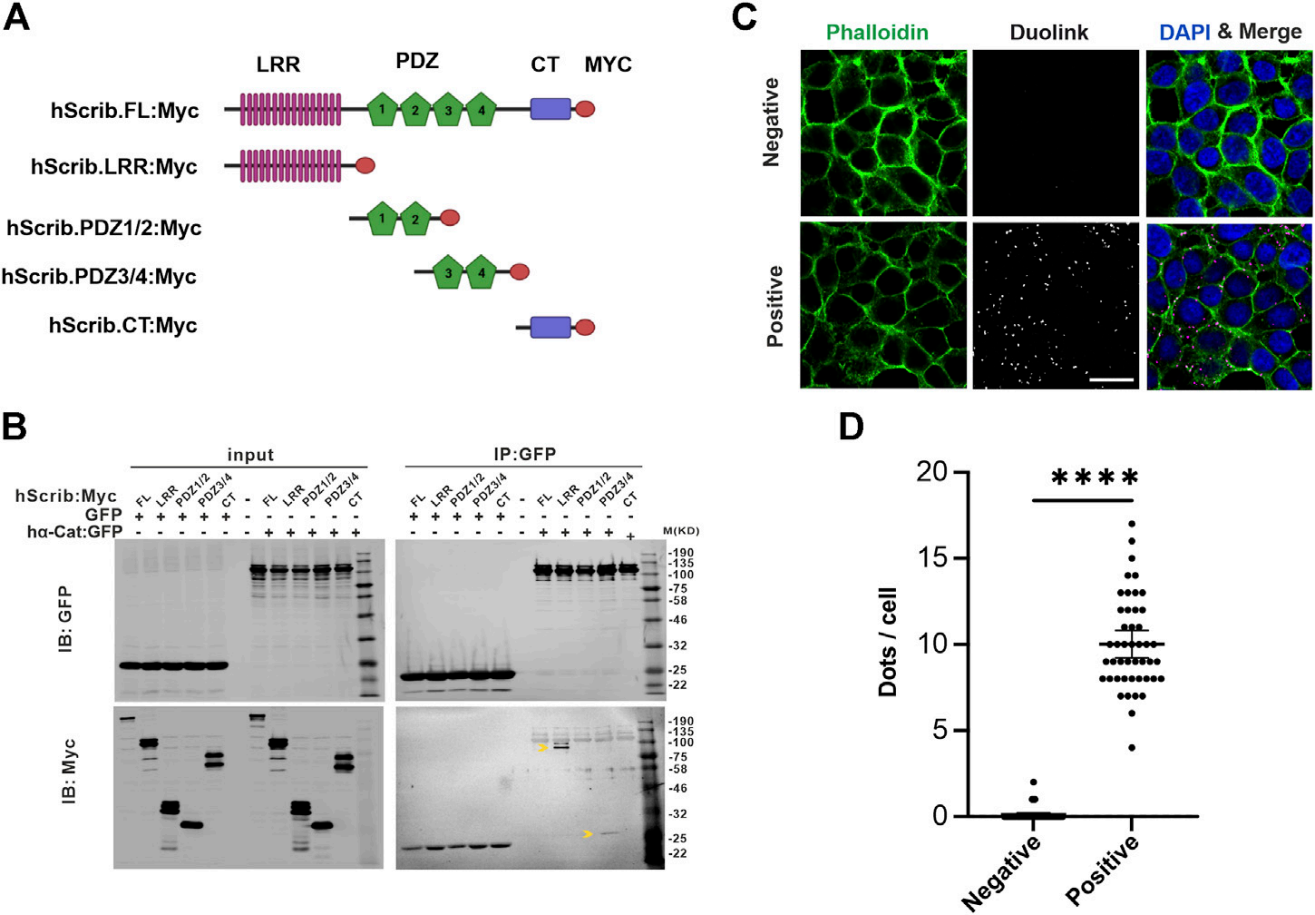
Scrib interacts with α-Cat in mammalian cells. **(A)** A schematic showing the structure of Scrib and fragments used in the immunoprecipitation assay. Scrib fragments include the leucine rich region (LRR), PDZ, and the C-terminal (CT) domains. **(B)** Co-Immunoprecipitation (Co-IP) and Western blotting assays suggest Scrib interacts biochemically with α-Cat through LRR and PDZ3/4 domains of Scrib. Arrowheads indicate LRR fragment and PDZ3/4 domain, respectively. **(C)** Staining of phalloidin (green) and Duolink Proximity Ligation Assays *in situ* in HEK293T cells. Scrib and α-Cat antibodies were co-incubated in experimental samples (Positive), whereas Myc and α-Cat antibodies were co-incubated in the controls (Negative). Bright dots (white, middle panel and magenta, right panel) in experimental samples show interaction between Scrib and α-Cat at endogenous expression levels. **(D)** Quantification of dots in each cell between control and experimental samples in **(C)**. n = 45. ****p < 0.0001. Data are means ±95% confidence intervals (CIs). Statistical significance was calculated by the two-tailed *t*-test. Scale bars, 20 μm **(C)**.

To understand whether the interaction between Scrib and α-Cat is also observed in vertebrates, we employed a mammalian cell culture system. HEK293T cells, derived from human embryonic kidney epithelium, were transfected with plasmids harboring tagged cDNAs of human Scrib (Scrib, Myc-tagged) and human α-Cat (α-Cat, GFP-tagged), or GFP as a negative control. Gene products were utilized in immunoprecipitations (IP) with antibodies directed against the Myc and GFP epitopes. To dissect functionally the domains of Scrib, various fragments containing different domains were co-expressed with α-Cat:GFP or GFP (Figure 1A). GFP or α-Cat:GFP interactors were precipitated using beads conjugated to a GFP-specific nanobody and subjected to Western blot assays. No Scrib or fragments thereof were detected in the GFP interactome, whereas LRR or PDZ3/4 domains of Scrib were immunoprecipitated by α-Cat:GFP, suggesting that the interaction between Scrib and α-Cat is conserved in vertebrates and invertebrates (Figure 1B). We barely detected full-length Scrib in the α-Cat:GFP interactome, suggesting that physical interactions with full length Scrib are transient or weak.

To further test protein interactions within the HEK293T cells, we employed proximity-ligation assay (PLA) to detect the endogenous interaction between Scrib and α-Cat *in situ* in cell culture (Alam, 2022). Rabbit anti-human Scrib and mouse anti-human α-Cat antibodies were combined to detect their interaction, and rabbit anti-Myc antibodies were used as a negative control when combined with anti-α-Cat antibodies. Only when two proteins are very proximal (40 nm or closer) to each other can they be ligated. Remarkably, multiple foci were observed in the cells incubated with antibodies against Scrib and α-Cat, in contrast to almost zero in the control (Figures 1C,D). Notably, these foci were mainly detected at the cell periphery, consistent with their cellular localization. These data further validate the interaction between Scrib and α-Cat in HEK293T cells.

### Scrib and α-cat are required for YAP suppression when cells become confluent

Next, we investigated the roles of Scrib and α-Cat interaction. In A375 cells the nuclear translocation of YAP is significantly reduced when cells become confluent (Fig. S1A, B). Both α-Cat and Scrib have been shown to be involved in YAP regulation in mammalian cells (Silvis et al., 2011; Totaro et al., 2018). To interrogate whether Scrib can regulate YAP through α-Cat, we performed RNAi-mediated knockdown (KD) of Scrib or α-Cat in confluent A375 cells. Both Scrib and α-Cat KD significantly increase the nuclear translocation of YAP (Fig. S1C-E). Furthermore, YAP protein signal is higher upon reduction of α-Cat or Scrib (Fig. S1C, D). This can be interpreted as cytosolic retention of YAP, ultimately leading to its degradation, which can be avoided when released upon knockdown of α-Cat or Scrib. To further understand how Scrib and α-Cat interact in A375 cells, we employed the Duolink PLA assay. Multiple foci were observed in the cells incubated with antibodies against Scrib and α-Cat (Fig.S1F, G). These data further support that the interactions between Scrib and α-Cat are conserved in mammalian epithelial cells.

### Exogenous scrib suppresses nuclear YAP translocation with α-cat

**FIGURE 2:**
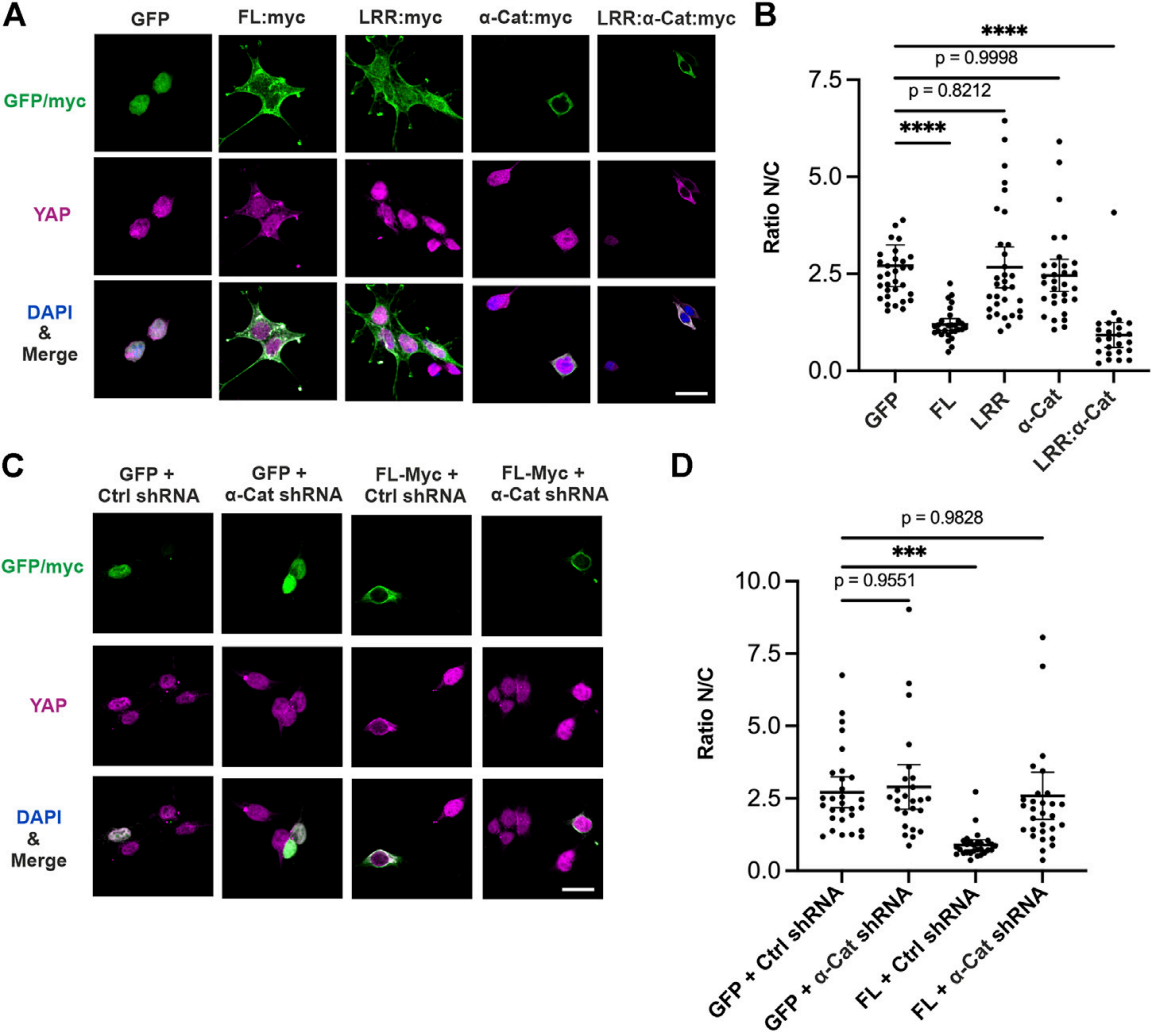
Scrib regulates YAP localization through interaction with α-Cat. **(A–D)** Immunostaining of YAP (magenta), GFP/Myc (green) and DAPI (blue) at low density of HEK293T cells. **(A)** Overexpressed Scrib. FL (FL:Myc), Scrib-LRR (LRR:Myc), α-Cat (αCat:Myc), Scrib-LRR:α-Cat (LRR:α-Cat:Myc), or GFP as control. Overexpression of Scrib. FL and Scrib-LRR:α-Cat leads to reduce the nuclear translocation of YAP in cells grown at low density, whereas Scrib-LRR domain or α-Cat alone does not. **(B)** Quantification of ratio of nuclear/cytoplasmic (N/C) YAP localization in **(A)**. *n* = 32. ****p < 0.0001. Data are means ±95% confidence intervals (CIs). Statistical significance was calculated by Dunnett’s test after one-way ANOVA. **(C)** Overexpressed Scrib. FL (FL:Myc) or GFP under α-Cat shRNA KD or control shRNA KD (ctrl shRNA) conditions. **(D)** Quantification of ratio of nuclear/cytoplasmic (N/C) YAP localization in **(C)**. *n* = 30. ***p = 0.0001. Data are means ±95% confidence intervals (CIs). Statistical significance was calculated by Dunnett’s test after one-way ANOVA. Scale bars, 20 μm **(A,C)**.

We next address how Scrib is involved in YAP localization while HEK293T cells are maintained at low density. Ectopic expression of Scrib is sufficient to suppress the nuclear translocation of YAP in these cells (Figures 2A,B). To further understand the mechanism by which Scrib regulates YAP, the LRR domain of Scrib, α-Cat or Scrib-LRR:α-Cat, a chimera comprising the Scrib LRR domain and α-Cat, were expressed. Expression of LRR domain or α-Cat barely affects ratio of nuclear/cytoplasmic (N/C) YAP localization. Remarkably, cells with Scrib-LRR:α-Cat expression show lower nuclear translocation of YAP (Figures 2A,B). We then wondered whether α-Cat is required for Scrib-mediated reduced nuclear localization of YAP. To test this idea, ectopic expression of Scrib and KD of α-Cat were combined. The ratio of N/C of YAP is largely reversed upon KD of α-Cat, suggesting that α-Cat is indispensable for Scrib-mediated YAP suppression (Figures 2C,D). Taken together, these data demonstrate that association with α-Cat is important for Scrib’s role in YAP suppression, and Scrib can regulate YAP through α-Cat.

### Spatiotemporal expression of scrib is tightly regulated in the *Drosophila* wing imaginal disc

**FIGURE 3:**
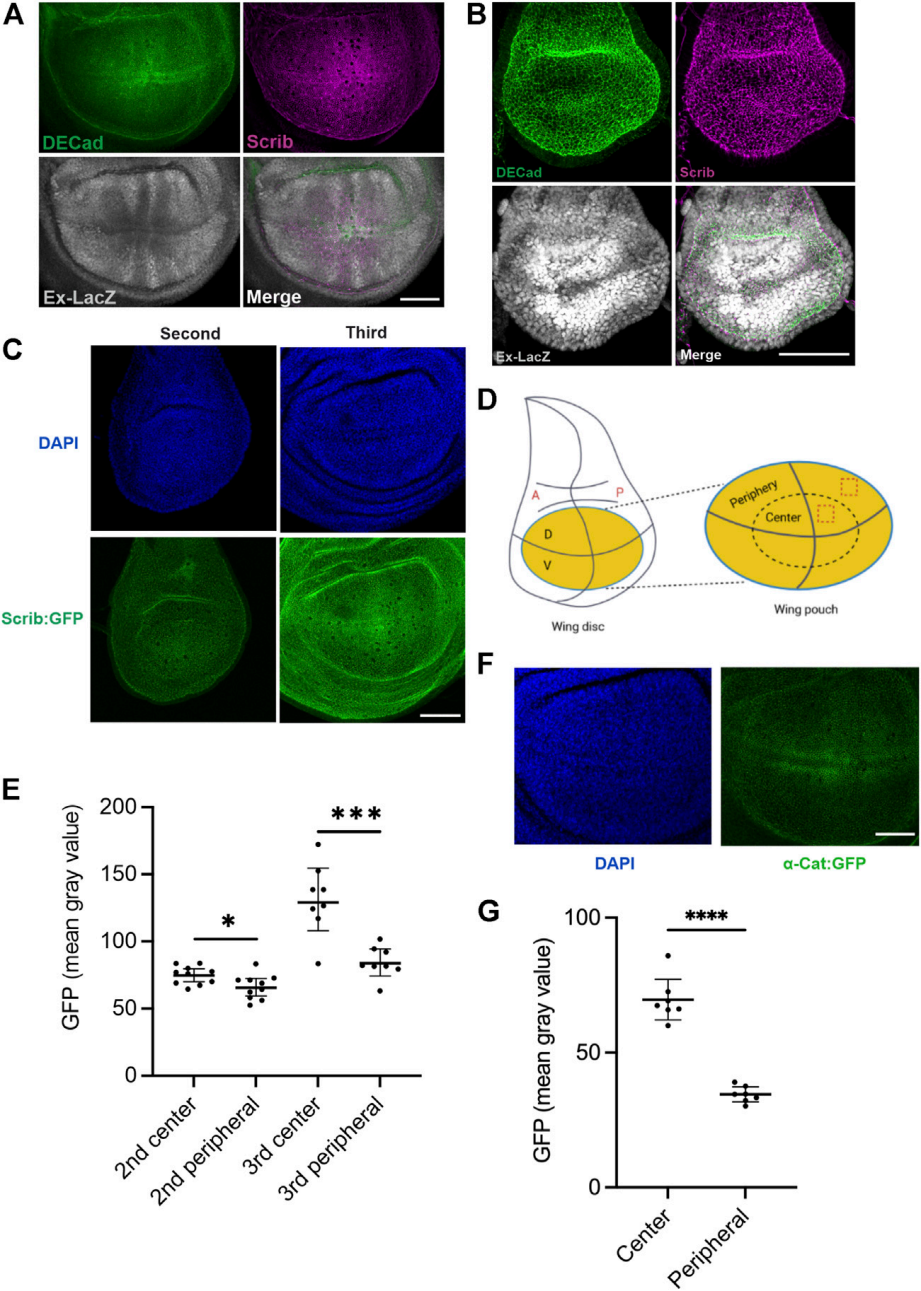
Scrib expression is complementary to Yki signal in *Drosophila* wing imaginal disc. **(A)** Wing imaginal disc in the third instar larval stage stained with DECad (green), Scrib (magenta) and Ex-lacZ (Yki activity, white). **(B)** Wing imaginal disc in the second larval stage stained with DECad (green), Scrib (magenta) and Ex-lacZ (white). Note that Yki activity indicated by Ex-LacZ is ubiquitous in the wing pouch. **(C)** DAPI (blue) and Scrib:GFP (green) images in the second and third instar larva. Images have been taken at the identical conditions. **(D)** Schematics of the *Drosophila* wing imaginal disc. Yellow region shows the wing pouch (left). Enlarged wing pouch is shown in right panel. The horizontal line indicates the dorsal **(D)** -ventral (V) compartment boundary, and the vertical line the anterior **(A)** -posterior (P) compartment boundary. Dashed boxes in the central and peripheral regions of the wing pouch are used for quantification of GFP intensity in **(C,F)**. **(E)** Quantification of fluorescent intensity (mean gray value) of central and peripheral region in **(C)**. *n* = 8. *p = 0.0255, ***p = 0.0004. Data are means ±95% confidence intervals (CIs). Statistical significance was calculated by the two-tailed *t*-test. **(F)** DAPI (blue) and α-Cat:GFP (green) images in the third instar larva. Images have been taken at the identical conditions for quantification. **(G)** Quantification of fluorescent intensity (mean gray value) of central and peripheral region in **(F)**. *n* = 7. ****p < 0.0001. Data are means ±95% confidence intervals (CIs). Statistical significance was calculated by the two-tailed *t*-test. Scale bars, 50 μm **(A–C)**, 40 μm **(F)**.

We then hypothesized that spatiotemporal control of Scrib expression plays an important role in epithelial tissue development. To understand how Scrib expression is regulated in developing tissues, an *in vivo* model is crucial. By employing the *Drosophila* larval wing imaginal disc, an exhaustively studied *in vivo* system for understanding epithelial homeostasis and growth, we investigated protein distributions of Scrib and E-Cad, a key component of AJs. Both Scrib and E-Cad show similar spatial distributions in the late third instar wing imaginal disc, when mitosis gradually decreases, with lower peripheral and higher medial expression (Figure 3A) (Pan et al., 2018). Intriguingly, these patterns are complementary to Yki signal, for which Ex-lacZ expression is a readout (Figure 3A) (Pan et al., 2018). We then analyzed Scrib and E-Cad expression in the second instar wing disc, in which mitosis is very active and ubiquitously observed in the wing pouch (Pan et al., 2018). Yki signal is ubiquitously expressed adjacent to the compartment boundary, and expression of both Scrib and E-Cad remains ubiquitous (Figure 3B). To quantify the Scrib expression level at different stages, we used GFP trap lines (Scrib:GFP) in which *scrib* expression is under the endogenous *scrib* enhancer (Kelso et al., 2004; Pan et al., 2018). GFP signal was collected under identical conditions to compare signaling intensities. Our results reveal that Scrib expression in the medial of the wing pouch increases at the late third instar in correspondence with down-regulation of Yki activity (Figures 3C–E). We then wondered how α-Cat expression is regulated. By using GFP trap lines (α-Cat:GFP) in which *α-cat* expression is under the endogenous *α-cat* enhancer, GFP signal was quantified in different positions. α-Cat expression is higher in the center than the periphery of the wing pouch at the late third instar (Figures 3F,G). Taken together, these data reveal that spatiotemporal expression of Scrib and α-Cat is tightly controlled in developing epithelial tissue.

### Quantitative control of scrib sufficiently regulates tissue growth in wing imaginal disc

**FIGURE 4:**
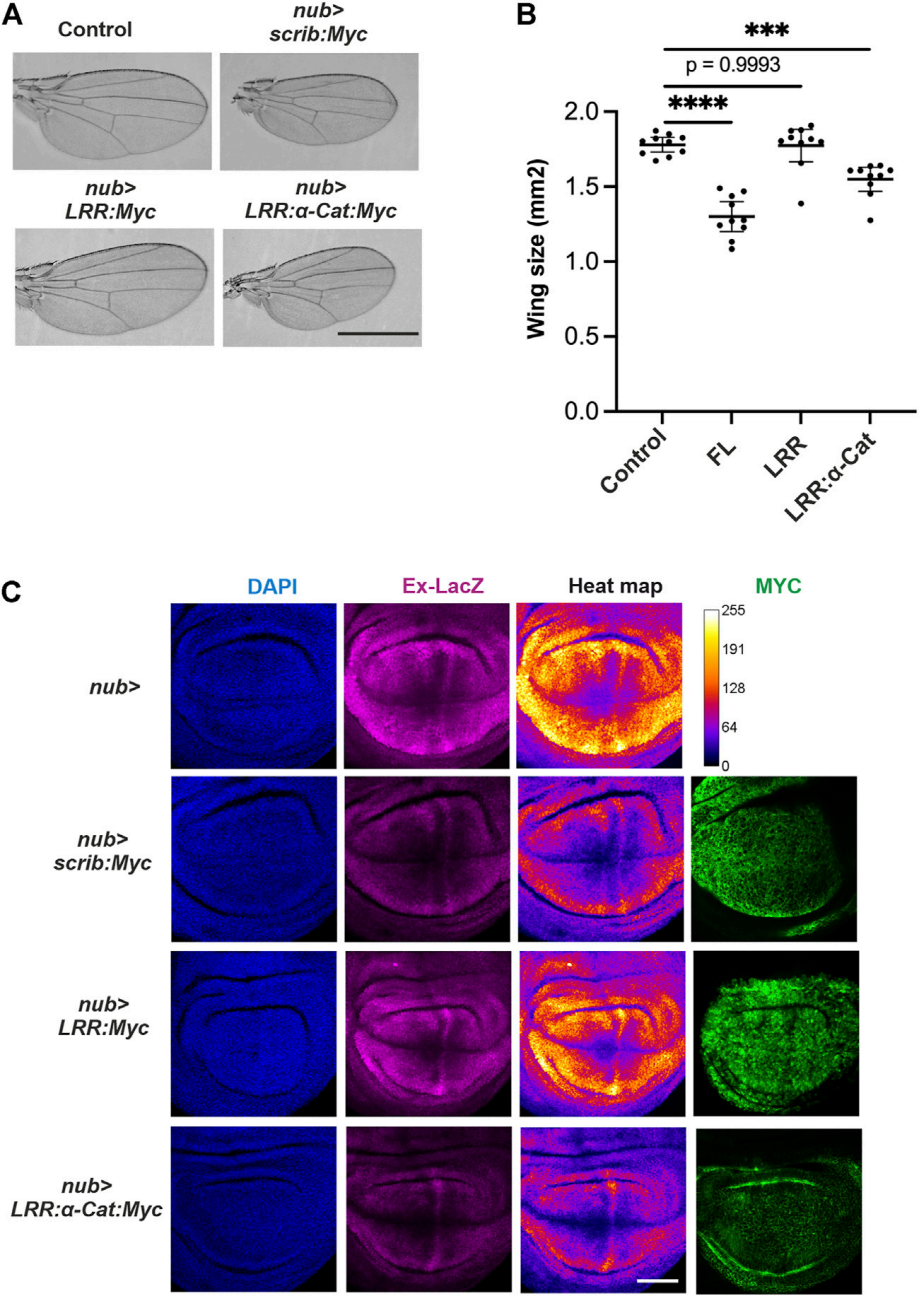
Scrib suppresses Yki activity in *Drosophila* wing. **(**A) Adult wings in control, *nub-GAL4>scrib.FL (nub > scrib:Myc)*, *nub-GAL4>scrib-LRR (nub* *>* *LRR:Myc)* and *nub-GAL4>scrib-LRR:αCat* chimera (*nub > LRR-αCat:Myc*). Full length Scrib and Scrib. LRR:αCat lead to smaller adult wings, Scrib-LRR domain has no effect. **(B)** Quantification of wing size in **(A)**, *n* = 20. ***p = 0.0005, ****p < 0.0001. Data are means ±95% confidence intervals (CIs). Statistical significance was calculated by Dunnett’s test after one-way ANOVA. **(C)** Wing imaginal disc stained with DAPI (blue), Ex-LacZ (magenta) and Myc antibody (green). Heat map showing intensities of LacZ staining. A scale for the heat maps is indicated on the right (top row). The heat map was produced using the ROI color coder plugin, matching measurements to a color of a lookup table (LUT) of ImageJ/FIJI. Yki activity is suppressed upon Scrib. FL and Scrib-LRR:αCat expression in the wing disc. Protein levels of Scrib. FL, Scrib-LRR and Scrib-LRR: αCat are visualized by Myc antibody staining (right panel). Note that Scrib-LRR protein appears to be more stable than Scrib. FL and Scrib-LRR: αCat proteins. Scale bars, 1 mm **(A)**, 50 μm **(C)**.

We next hypothesized that the amount of available Scrib is crucial for growth control in wing development. If this is the case, ectopic expression of Scrib alone may change the tissue size through modulating Yki activity. By employing an UAS-GAL4 system in *Drosophila*, Scrib or related proteins were overexpressed in the developing wing. Ectopic expression of Scrib or Scrib-LRR:α-Cat chimera, but not Scrib-LRR, resulted in a significantly smaller wing (Figures 4A,B). These results suggest that amount of Scrib functions as a growth signal. To understand the molecular mechanisms underlying this, we studied the wing imaginal disc. Our data reveal that ectopic expression of Scrib or Scrib-LRR:α-Cat chimera, but not Scrib-LRR, significantly suppresses Yki acvitity (Figure 4C). Since all the UAS transgenic flies were generated by the site-specific insertion system, the transcriptional level among different constructs are ensured to be equivalent (Gui et al., 2021). Accordingly, protein levels of different transgenic lines reflect protein stability (Figure 4C). These results suggest that Yki signal is regulated by amounts and quality of Scrib-related proteins.

### Scrib and α-cat interactions sustain tissue growth and homeostasis in wing imaginal disc

**FIGURE 5:**
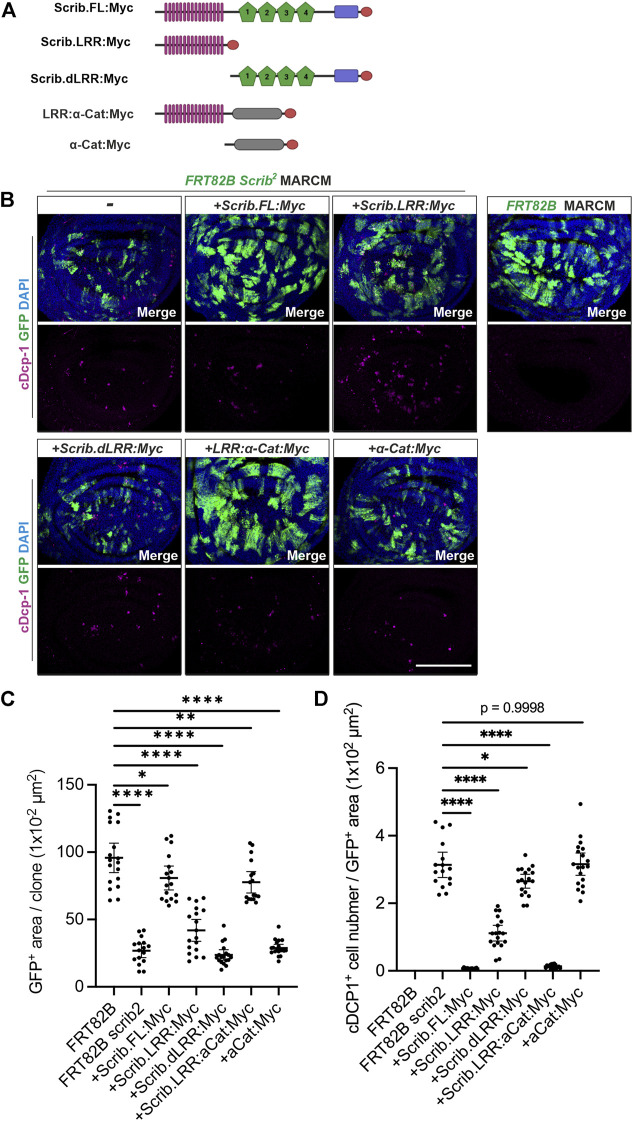
Scrib and α-Cat interactions sustain tissue growth and homeostasis in *Drosophila* wing imaginal disc. **(A)** Schematics show transgenic constructs with Myc tag at C-terminus, used in immunostaining analysis. **(B)** MARCM analysis shows the capability of various forms of Scrib proteins in rescuing the clonal, cell viability of *scrib* null (*scrib* ^*2*^) mutant cells labelled with mCD8:GFP (GFP^+^, green). Immunostaining of cleaved Dcp-1 (cDcp-1, an apoptotic marker, magenta). **(C)** Quantification of GFP^+^ area in **(B)**. *p = 0.0147, **p = 0.0019, ****p < 0.0001. Data are means ±95% confidence intervals (CIs). Statistical significance was calculated by Dunnett’s test after one-way ANOVA. **(D)** Quantification of apoptotic cells (marked by cDcp-1) in **(B)**. *p = 0.0167, ****p < 0.0001. Data are means ±95% confidence intervals (CIs). Statistical significance was calculated by Dunnett’s test after one-way ANOVA. Scale bar, 100 μm **(B)**.

To further address how the Scrib/α-Cat complex contributes to tissue growth and homeostasis, we employed mosaic analysis with a repressible cell marker (MARCM) clone analysis in the wing imaginal disc (Lee and Luo, 1999). We applied MARCM analysis by expressing various forms of Scrib in *scrib* null mutant (*scrib* ^*2*^) clones (Figure 5A). It has been shown that cell competition takes place when *scrib* ^*2*^ clones were generated in the wing imaginal disc (Chen et al., 2012). Since JNK signal was up-regulated in *scrib* ^*2*^ clones to suppress Yki activity, apoptosis (marked by cDcp1) but not over proliferation was observed in *scrib* ^*2*^ clones, thus the sizes of *scrib* ^*2*^ clones are significantly smaller than control cells, (Figures 5B–D). These phenotypes are largely restored by expression of full-length Scrib (Figures 5B–D). Scrib-LRR partially restores clone sizes and reduces numbers of apoptotic cells, but with lower efficiency than full-length Scrib (Figures 5B–D). Intriguingly, clone sizes and number of apoptotic cells are fully rescued by expression of Scrib-LRR:α-Cat chimera protein (Figures 5B–D). Expression of deleted LRR-Scrib or α-Cat alone shows similar phenotypes to *scrib* ^*2*^ clones (Figures 5B–D). Taken together, these results indicate that the LRR-Scrib and α-Cat complex sufficiently replaces full-length Scrib signaling for tissue growth and homeostasis when those cells are surrounded by wild type cells.

## Discussion

In this work, we show that interactions of Scrib and α-Cat are conserved between *Drosophila* and human cells. As a key epithelial cell polarity determinant, Scrib is crucial in preserving epithelial integrity, since its loss gives rise to neoplasia (Bilder, 2004; Zeitler et al., 2004; Hariharan and Bilder, 2006; Bonello and Peifer, 2019; Khoury and Bilder, 2020). Therefore, other Scrib functions are difficult to determine due to cell death and tissue disorganization. Our recent study reveals that α-Cat, one of the core components of AJs, interacts with Scrib to regulate tissue growth in *Drosophila* wing imaginal disc, in addition to regulating apico-basal polarity, in a non-autonomous manner (Gui et al., 2021). Here, we expand our study to mammalian cells to investigate whether similar relationships operate in mammalian cells. We then further hypothesized that control of Scrib levels is a means of regulating tissue growth, and provided evidence that this is the case in *Drosophila* wing development.

To characterize how Scrib and α-Cat regulate YAP localization in a context-dependent manner, we used the human HEK293T cell line for its ease of use in gene transfection and knockdown studies. Ectopic expression of Scrib sufficiently reduces YAP localization in the nucleus (Figure 2). Importantly, α-Cat is needed for Scrib-mediated YAP localization, but ectopic expression of α-Cat alone is not sufficient for such a change. Instead, a Scrib-LRR:α-Cat chimera effect YAP localization (Figure 2). Since the LRR domain of Scrib is considered to be key for cortical localization (Zeitler et al., 2004) and Scrib and α-Cat are co-localized in mammalian cells (Figure 1), we consider that Scrib has a conserved mechanism to regulate YAP activity in co-operation with α-Cat.

One of the novel findings in this study is that quantitative regulation of Scrib protein levels is itself a functional regulator of growth control. Ectopic expression of Scrib in *Drosophila* wing imaginal disc sufficiently down-regulates Yki signal, leading to smaller tissue size of the adult wing (Figures 4A,B). To maintain homeostasis of epithelial cells, Scrib complex is one of the basic elements (Thompson, 2013), thus loss of Scrib complex naturally causes loss of epithelial homeostasis. By using a conditional gene knockdown approach, we previously showed that Scrib and BMP signaling form a feedforward loop to regulate BMP signal in *Drosophila* pupal wing for wing vein differentiation, which is distinct from cell polarity maintenance (Gui et al., 2016). Since our results showed that Scrib expression is indeed dynamically regulated, showing complementary patterns to Yki activity (Figure 3), we presume that similar feedback/feedforward mechanisms are utilized for growth control. Although how Scrib expression is regulated in a quantitative manner in growing tissue remains to be addressed, future study will reveal relevant molecular mechanisms in greater detail, and how this unique mechanism contributes to epithelial tissue growth.

Finally, we confirmed that chimeric protein Scrib-LRR:α-Cat is functional *in vivo*. As previously described, *scrib* mutant clones in wing imaginal disc are efficiently eliminated through oncogenic cell competition, although *scrib* mutant cells are intrinsically oncogenic (Chen et al., 2012; Kanda and Igaki, 2020). Smaller *scrib* mutant clone size and increased apoptosis provide evidence for this phenomenon (Figure 5). Our data reveal that full-length Scrib, as well as Scrib-LRR:α-Cat chimera, sufficiently rescue this phenotype (Figure 5). In contrast, neither Scrib-LRR nor α-Cat alone is sufficient for rescue. These results further support the physiological significance of α-Cat, which cooperates with Scrib *in vivo* for growth regulation and homeostasis of epithelial tissues.

In summary, this study reveals that interactions of Scrib and α-Cat are conserved between human and *Drosophila* cells to play a role in growth and homeostasis of epithelial tissues. Regulating Scrib expression levels in developing tissues is one of the means of growth control. Future studies will unveil further implications of Scrib and α-Cat interactions in development of epithelial tissues and human diseases.

## Data Availability

The original contributions presented in the study are included in the article/Supplementary Material further inquiries can be directed to the corresponding author.
