# Supplementary material for: Scribble and α-Catenin cooperatively regulate epithelial homeostasis and growth
Source: Front Cell Dev Biol. 2022 Sep 21;10:912001. doi: 10.3389/fcell.2022.912001 (PMC9532510; doi:10.3389/fcell.2022.912001)
Supplement: Supplementary file 1 [file DataSheet1.PDF]

Supplementary Material

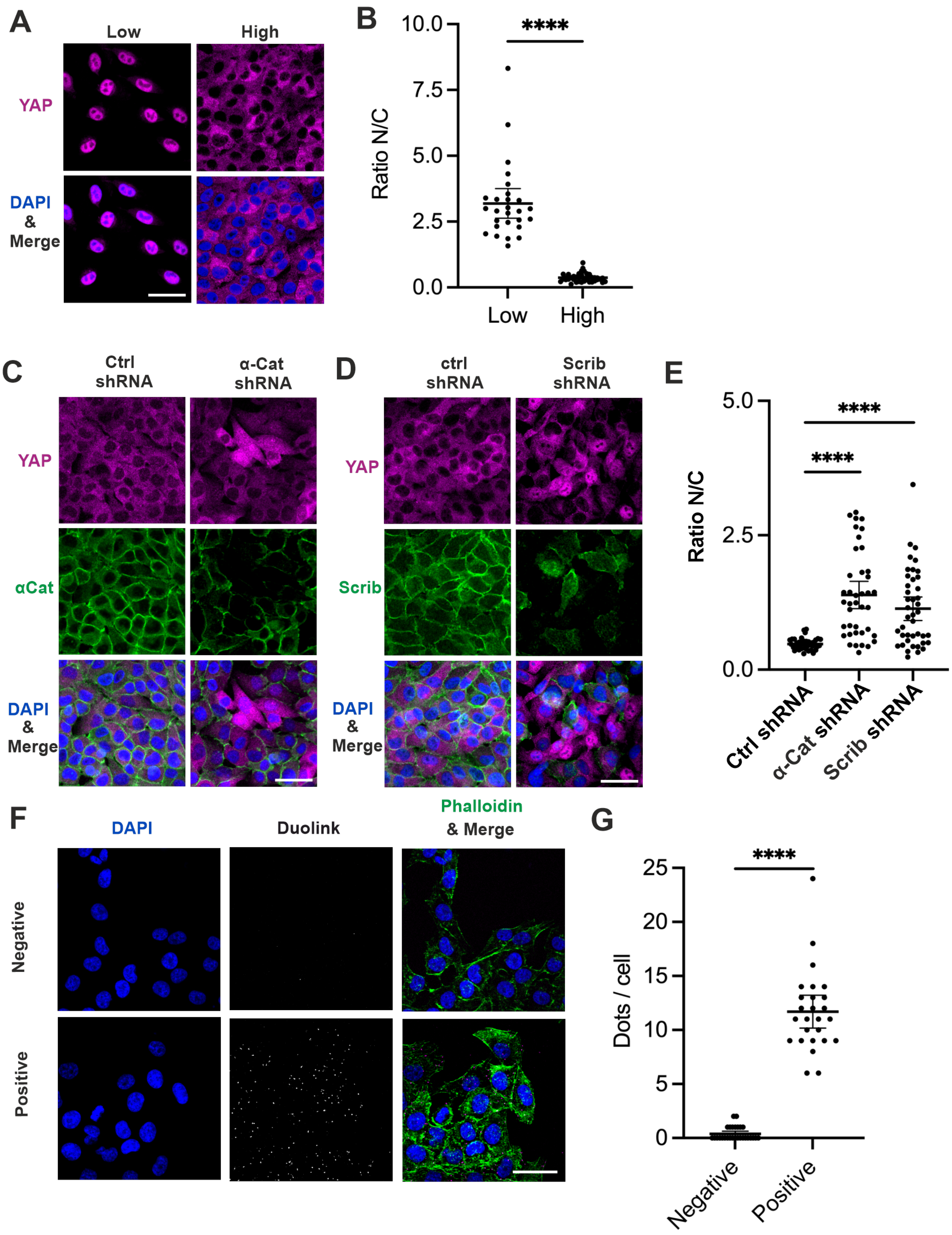
A
Low
High
YAP
DAPI
&
Merge
B
10.0
7.5
5.0
2.5
0.0
Ratio N/C
****
Low
High
C
Ctrl
shRNA
α-Cat
shRNA
YAP
αCat
DAPI
&
Merge
D
ctrl
shRNA
Scrib
shRNA
YAP
Scrib
DAPI
&
Merge
E
5.0
2.5
0.0
Ratio N/C
****
****
Ctrl shRNA
α-Cat shRNA
Scrib shRNA
F
DAPI
Duolink
Phalloidin
& Merge
Negative
Positive
G
25
20
15
10
5
0
Dots / cell
****
Negative
Positive

Figure S1. Scrib and α-Cat affect YAP localization in human A375 cells. (A) Immunostaining of YAP in A375 cells. At low cell density, YAP (magenta) is localized in the nuclei of cells. In contrast, YAP translocates to the cytoplasm at high cell density. (B) Quantification of ratio of nuclear /cytoplasmic (N/C) YAP localization in (A). n=27. ****P < 0.0001. Data are means ± 95 % confidence intervals (CIs). Statistical significance was calculated by the two-tailed *t*-test. (C) Staining of YAP (magenta) and α-Cat (green) at high A375 cell density in control (ctrl shRNA) and α-Cat knockdown (α-Cat shRNA). Knockdown of α-Cat leads to nuclear YAP localization. (D) Staining of YAP (magenta) and Scrib (green) at high cell density in control (ctrl shRNA) and Scrib knockdown (Scrib shRNA). Knockdown of Scrib affects YAP localization at high cell density. (E) Quantification of ratio of nuclear /cytoplasmic (N/C) YAP localization in (C) and (D). n=40. ****P < 0.0001. Data are means ± 95 % confidence intervals (CIs). Statistical significance was calculated by Dunnett's test after one-way ANOVA. (F) Staining of phalloidin (green) and Duolink Proximity Ligation Assays in situ in A375 cells. Scrib and α-Cat antibodies were co-incubated in experimental samples (Positive), whereas Myc and α-Cat antibodies were co-incubated in the controls (Negative). Bright dots (white, middle panel and magenta, right panel) in experimental samples show interaction between Scrib and α-Cat at endogenous expression levels. (G) Quantification of dots in each cell between control and experimental samples in (F). n=26. ****P < 0.0001. Data are means ± 95 % confidence intervals (CIs). Statistical significance was calculated by the two-tailed *t*-test. Scale bars, 50 µm (A, C, D), 40 µm (F).
